# Temperature-Controlled Crystal Size of Wide Band Gap Nickel Oxide and Its Application in Electrochromism

**DOI:** 10.3390/mi12010080

**Published:** 2021-01-14

**Authors:** Muyang Shi, Tian Qiu, Biao Tang, Guanguang Zhang, Rihui Yao, Wei Xu, Junlong Chen, Xiao Fu, Honglong Ning, Junbiao Peng

**Affiliations:** 1 State Key Laboratory of Luminescent Materials and Devices, Institute of Polymer Optoelectronic Materials and Devices, South China University of Technology, Guangzhou 510640, China; 201430320229@mail.scut.edu.cn (M.S.); msgg-zhang@mail.scut.edu.cn (G.Z.); xuwei@scut.edu.cn (W.X.); msjlchen@gmail.com (J.C.); 201630343721@mail.scut.edu.cn (X.F.); psjbpeng@scut.edu.cn (J.P.); 2 Department of Intelligent Manufacturing, Wuyi University, Jiangmen 529020, China; timeqiu@hotmail.com; 3 Guangdong Provincial Key Laboratory of Optical Information Materials and Technology & Institute of Electronic Paper Displays, South China Academy of Advanced Optoelectronics, South China Normal University, Guangzhou 510006, China; biao.tang@guohua-oet.com

**Keywords:** nickel oxide, annealing temperature, crystallite size, optical band gap, electrochromic device

## Abstract

Nickel oxide (NiO) is a wide band gap semiconductor material that is used as an electrochromic layer or an ion storage layer in electrochromic devices. In this work, the effect of annealing temperature on sol-gel NiO films was investigated. Fourier transform infrared spectroscopy (FTIR) showed that the formation of NiO via decomposition of the precursor nickel acetate occurred at about 300 °C. Meanwhile, an increase in roughness was observed by Atomic force microscope (AFM), and precipitation of a large number of crystallites was observed at 500 °C. X-ray Diffraction (XRD) showed that the NiO film obtained at such a temperature showed a degree of crystallinity. The film crystallinity and crystallite size also increased with increasing annealing temperature. An ultraviolet spectrophotometer was used to investigate the optical band gap of the colored NiO films, and it was found that the band gap increased from 3.65 eV to 3.74 eV with the increase in annealing temperature. An electrochromic test further showed that optical modulation density and coloring efficiency decreased with the increase in crystallite size. The electrochromic reaction of the nickel oxide film is more likely to occur at the crystal interface and is closely related to the change of the optical band gap. An NiO film with smaller crystallite size is more conducive to ion implantation and the films treated at 300 °C exhibit optimum electrochromic behavior.

## 1. Introduction

**Figure 1:**
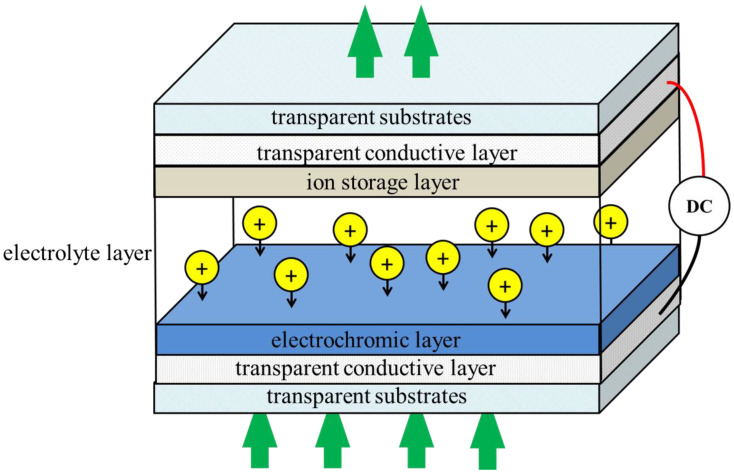
A typical ECD design. Arrows indicate the movement of ions and electrons in an applied electric field.

Nickel oxide (NiO) is an important wide band gap semiconductor material with a band gap width of approximately 3.6 to 4.0 eV. As one of the rare p-type semiconductors in transition metal oxides, nickel oxide has a stable band gap and excellent electrochromic properties [1]. As a new functional material, NiO has applications in many fields, such as a hole-transporting layer in solar cells [2,3], as a p-type transparent semiconductor [4,5], and as an electrochromic layer or an ion storage layer in electrochromic devices [6,7,8]. An electrochromic device (ECD) can adjust the modulating optical transmittance in the visible light region through ion implantation and extraction in response to the switching of an externally applied potential [9,10]. Figure 1 shows a generic ECD structure called a five-layer “battery-type” structure [11]. In the middle of two transparent substrates (generally a glass substrate), there are five superimposed thin layers, which are respectively an electrochromic layer, an ion storage layer, an electrolyte layer, and two transparent conductive layers. Energy-saving features include low power consumption, large optical modulation, high coloring efficiency, and good optical storage, making ECD useful for smart Windows, skylights, etc. [12,13]. Since the discovery of the electrochromic properties of NiO, extensive studies have been conducted on NiO-based electrochromic devices [14]. As one of the best anode electrochromic materials, NiO is often used to form complementary electrochromic devices with cathode electrochromic materials like WO_3_, and this type of electrochromic device has better optics modulation capability [15,16].

So far, various methods, include sputtering [17], chemical vapor deposition [18], electron-beam evaporation [19], and sol-gel [20] have been used for preparing NiO films. Currently, magnetron sputtering is used as a commercial technology because it can produce films with high uniformity and reliability. However, such a method requires expensive equipment. In contrast, the sol-gel method is much more desirable as far as the cost is concerned. More importantly, sol-gel technology is promising for future commercial device manufacturing via the low-cost printing route. However, at present, the sol gel prepared films still have problems like non-uniformity and poor repeatability. With the development of new sol-gel technology, such as inkjet printing [13], sol-gel technology is promising for commercial applications in the future.

In sol-gel technology, a key parameter is annealing temperature [21], which is critical in controlling the microstructure and electrochromic properties of the films. In this paper, the microstructure, electrical properties, optical modulation capabilities, and coloring efficiency of sol gel prepared nickel oxide films obtained under different annealing temperatures were analyzed, and the results may improve the current understanding of the relationship between the microstructure and performance of such oxide films.

## 2. Materials and Methods

Nickel acetate (C_4_H_6_NiO_4_, Macklin Biochemical Co. Ltd., Shanghai, China) was added to 2-Methoxyethanol (C_3_H_8_O_2_, Macklin Biochemical Co. Ltd., Shanghai, China) and mixed, then ethanolamine was added as a stabilizer, and the mixture was heated in an oil bath at 60 °C for 2 h. Finally, it was left to stand at room temperature for 24 h and aged to obtain sol-gel. Spin coating technology was used to prepare NiO films on commercial indium tin oxide (ITO) glass. The thickness of NiO is optimized and controlled by the solution concentration and spin coating parameters, which has an important impact on the electrochromic transmittance modulation ability. In this work, our study focuses on changing annealing temperature while keeping other variables consistent, such as sol concentration, spin coating parameters, substrate, and electrolyte. These deposited films were annealed at 100 °C, 200 °C, 300 °C, 400 °C, and 500 °C for 60 min.

The crystals of the film were analyzed by X-ray diffraction (XRD, PANalytical Empyrean DY1577, PANalytical, Almelo, The Netherlands). Surface morphology was measured with an atomic force microscope (AFM, Nano Instruments BY3000, Nano Instruments, Beijing, China). In the simple device constructed, a 0.5 mol/L KOH solution was used as an electrolyte, and ITO was used as an electrode to measure its electrochromic performance. The transmission spectrum was measured by an ultraviolet spectrophotometer (SHIMADZU UV2600, SHIMADZU, Tokyo, Japan) with ITO glass as a blank. The current of the electrochromic test was recorded by an electrochemical workstation (CH Instruments CHI600E, CH Instruments, Shanghai, China). 

## 3. Results

**Figure 2:**
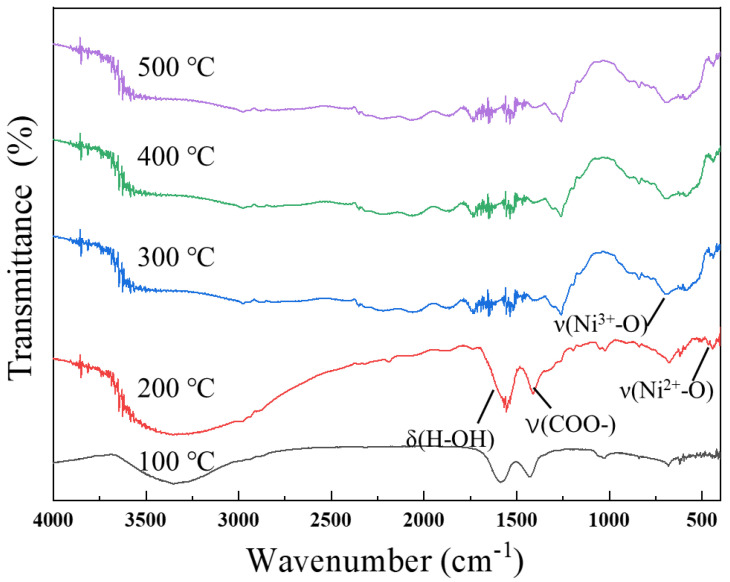
FTIR spectra of NiO films annealed at different temperatures.

The FTIR spectra of the films are shown in Figure 2. With increasing temperature, the absorption caused by acetate groups at 1465 cm^−1^ [22] and that due to the δ(H–OH) bonding at 1630 cm^−1^ [23] of the precursor gradually gets weak and even disappears at 300 °C, which indicates that the nickel acetate precursor has largely transformed to NiO at such a temperature. The FTIR spectra of NiO films annealed above 200 °C show that both ν (Ni^2+^–O) vibration at about 470 cm^−1^ and ν (Ni^3+^–O) vibration at about 470 cm^−1^ exist simultaneously, which indicates excess oxygen, and leads to a deviation of the stoichiometric ratio. This is a common phenomenon in the preparation of nickel oxide, which leads to lattice distortion and lattice constant changes. This is consistent with the results of lattice constants calculated by XRD below [24]. In addition, the FTIR spectra of the samples after annealing at 300 °C, 400 °C, and 500 °C show similar shapes, indicating that the chemical composition of the films was basically stable when the temperature was higher than 300 °C.

**Figure 3:**
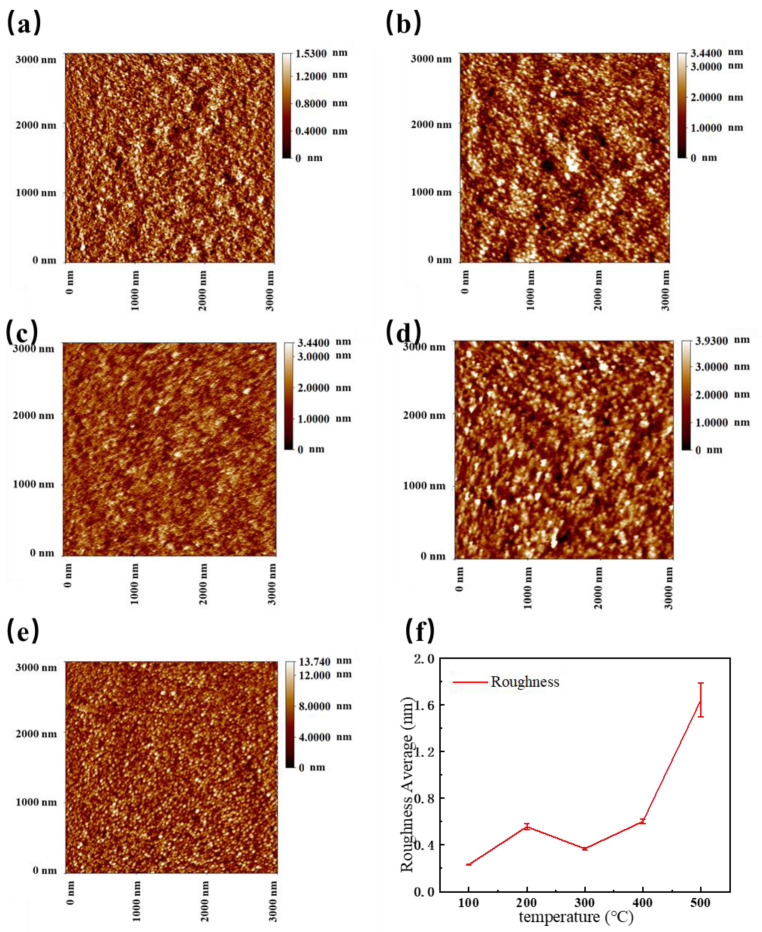
The atomic force microscope (AFM) images 3000 nm × 3000 nm and the roughness of NiO films. (**a**) 100 °C; (**b**) 200 °C; (**c**) 300 °C; (**d**) 400 °C; (**e**) 500 °C; (**f**) the roughness of NiO films, which are read by the support software of AFM.

**Figure 4:**
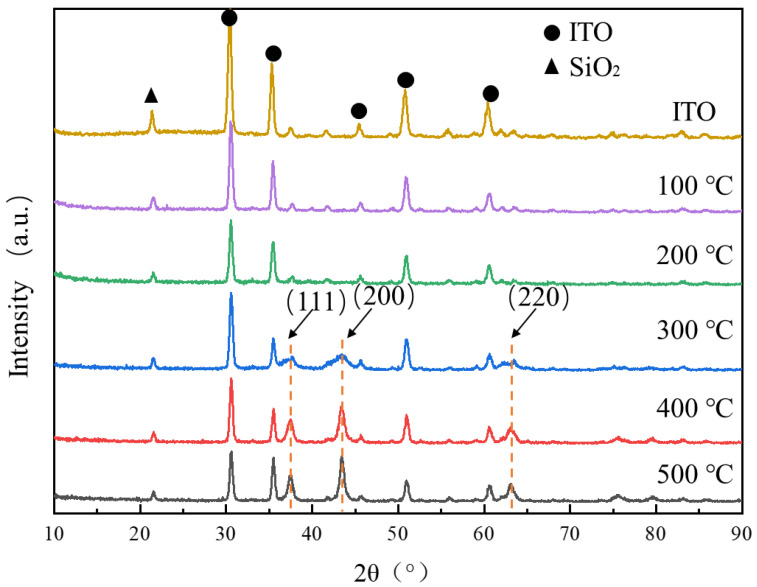
XRD patterns of NiO films annealed at different temperature.

The surface morphology of the films measured by AFM are shown in Figure 3a–e. Figure 3f shows a comparison of the roughness of the films. The surface of the NiO film annealed at 100 °C is the smoothest. This is because the solvent ethylene glycol methyl ether, with a boiling point of about 150 °C, still exists in the films. Probably due to the evaporation of the solvent at 200 °C, surface defects such as pores are formed and the sample annealed at 200 °C has a higher roughness than the films annealed at 100 °C. However, heat treatment at a higher temperature of 300 °C largely removes the defects and hence gives films with a reduced roughness, as shown in Figure 3c. As the heat treatment temperature is further increased, the roughness is increased again. This is mainly due to the increase in crystallinity and precipitation of relatively larger crystallites. This is partly consistent with the increased diffraction intensity, as shown in XRD observation (Figure 4). 

Figure 4 shows the XRD patterns of films annealed at different temperatures and ITO substrates.

The crystal structure of these films was further analyzed by HighScore Plus. The XRD patterns of the samples after annealing at 100 °C and 200 °C show similar shapes with the XRD pattern of ITO glass. Based on the information obtained from the FTIR spectrum, it can be judged that nickel oxide has not yet formed at this stage. Furthermore, there are three diffraction peaks (111) (200) (220), which match with standard card of nickel oxide (Reference code: 01-071-1179 by HighScore Plus 3.0e) of NiO at the patterns of the NiO films annealed at 300 °C, 400 °C, and 500 °C, which demonstrate that these films began to crystalline when the annealing temperature is higher than 300 °C [25]. All three diffraction peaks show a broadening phenomenon, which is caused by insufficient crystallinity or too small crystallite size. As the annealing temperature increased, the three diffraction peaks at the patterns of the sample became sharper, which represent an increase in crystallinity and crystallite size.

In order to measure the degree of crystallinity, we calculated the crystallite size and lattice parameter in different crystal plane directions of NiO films annealed at different temperatures. The lattice parameter could be calculated by using the following equation:

(1) $$a=\frac{\lambda }{2sin\theta \sqrt{h^{2}+k^{2}+l^{2}}}$$

Crystallite size could be calculated by measuring the half-maximum value of the X-ray diffraction peak (FMHM) using the Debye-Scherrer formula [26]:

(2) $$D=\frac{K\lambda }{\beta \cos\theta }$$

Among them, *D* is the average thickness (nm) of the crystal crystallites in the direction perpendicular to the crystal plane; *K* is the Scherrer constant, which is 0.89 in spherical particles, 0.94 in cubic particles, and nickel oxide crystals are cubic structures, so *K* here is 0.94; *h, k* and *l* are the Miller index of the corresponding crystal orientation; *λ* is the X-ray wavelength, 0.154056 nm; *β* is the half-width of the X-ray diffraction peak (FMHM), *θ* is the diffraction angle, and the values of *β* and *θ* are obtained by HighScore Plus 3.0e.

The lattice parameters of the nickel oxide films at different annealing temperatures in each crystal orientation were shown in Table 1.

As the annealing temperature increases, the lattice constant in the (111) and (220) directions decrease (approximately from 0.417 nm to 0.416 nm), while the lattice constant in the (200) direction hardly changes (about 0.416 nm). The lattice constant of the nickel oxide crystal is about 0.416 nm [27,28], and the lattice constant of the (111) and (220) crystal directions of the nickel oxide annealed at 300 °C is slightly higher than this value, which may be caused by lattice distortion. As the annealing temperature increases, the defects in the crystal decrease, so the lattice parameters of each crystal orientation gradually approach 0.416 nm.

In Table 2, it is observed that the crystallite size increased with increasing annealing temperature. The crystallite boundaries between the crystallites can be regarded as a type of defect, and the annealing process improves the lattice energy and can reduce the defects, thereby promoting the growth of the crystal crystallites. Therefore, as the temperature increases, more energy is provided during the annealing process. The crystallinity of the crystallites increased, and the crystallites grew larger, which is consistent with the above analysis of the lattice parameters.

The band gap of the NiO film can be measured and analyzed by an ultraviolet spectrophotometer. The optical band gap is distinguished from the band gap measured by other methods. According to Equation (3), the optical band gap can be calculated [29] as:

(3) $$\alpha hv=A{\left(hv-Eg\right)}^{n}$$

where α is the absorption coefficient, which can be measured by the ultraviolet spectrophotometer; *h* is the Planck constant; n is the light frequency; A is a proportionality constant; *Eg* is the optical band gap; and *n* is a number which is 1/2 for the direct band gap semiconductor and 2 for the indirect band gap semiconductor. In this work, *n* is 1/2 because NiO was a direct semiconductor.

To further investigate the electrochromic effects on the optical band gap of the NiO film, the optical band gap of the NiO film in a bleached state and colored state were analyzed. The electrochromic test was performed on NiO with ITO as the cathode and anode in a 0.5 mol/L KOH solution, and the applied potential was ± 2.5 V. During the electrochromic coloration and bleaching processes, the following electrochemical reactions take place in NiO layers [24,29]:

(4) $$\left(\mathrm{Bleached}\right)NiO+OH^{-}\underset{\;}{↔}\left(\mathrm{Colored}\right)NiOOH+e^{-}$$

(5) $$\left(\mathrm{Bleached}\right)Ni{\left(OH\right)}_{2}\underset{\;}{↔}\left(\mathrm{Colored}\right)NiOOH+H^{+}+e^{-}$$

**Figure 5:**
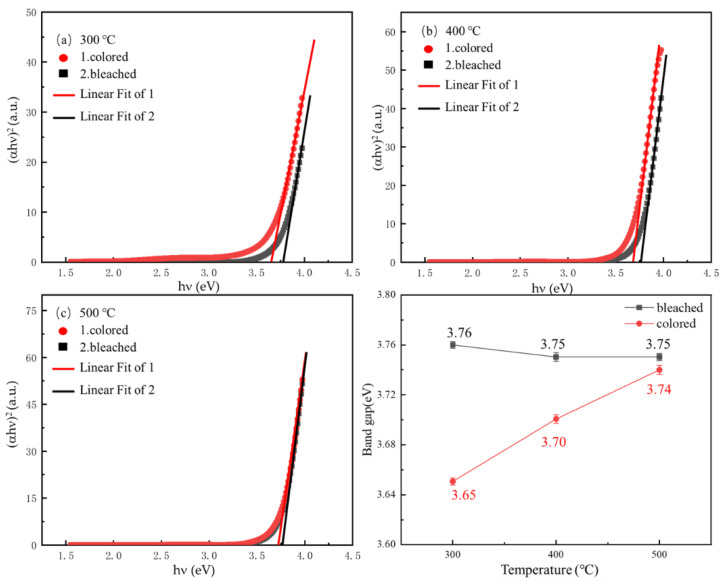
Optical band gap energy of NiO films in a colored state and bleached state. (**a**) 300 °C; (**b**) 400 °C; (**c**) 500 °C; and (**d**) a comparison of optical band gap energy of NiO films annealed at different temperatures and electrochromic states (colored and bleached).

Figure 5a–c illustrate the relationship between *(αhν)^2^* and photon energy *hν*. These curves are calculated using the transmission spectra of the NiO film in the colored state and the bleached state. For example, it can be extracted by the start of the optical transition of the NiO film near the band edge, which is equal to the value of the intercept of the fitted line. Figure 5d shows the comparison of the optical band gap values of NiO films annealed at different temperatures and electrochromic states (colored and bleached), indicating that as the annealing temperature increases, the *Eg* of the colored (initial state) NiO film changes; 3.65 eV increased to 3.74 eV, while the band gap of the bleached NiO film hardly changed.

Coloring efficiency (CE (η)) determines the amount of change in optical density (∆OD) based on the injected/injected charge (Qi) at a specific wavelength, that is, the amount of charge required to maintain optical density. It is given by [30]:

(6) η=(ΔODQi)=(lg(TbTc)Qi)

**Figure 6:**
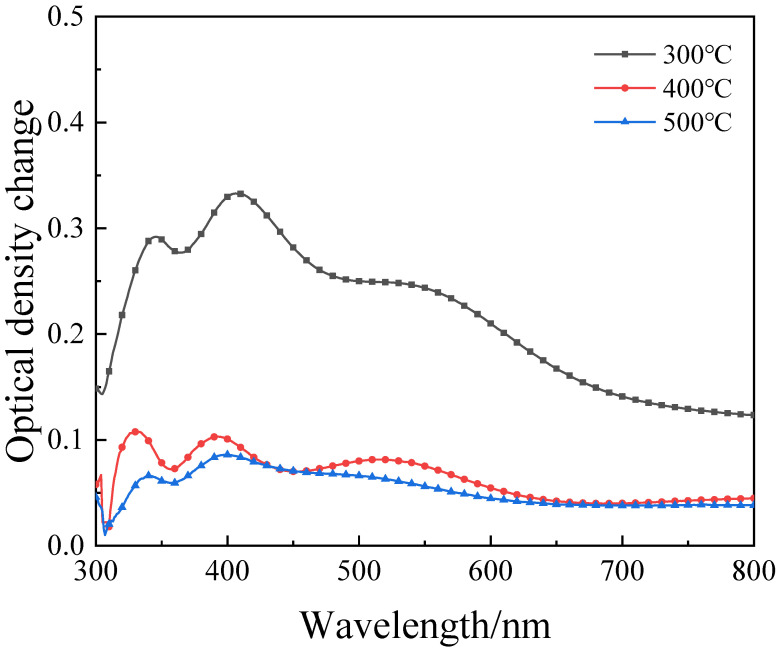
ΔOD of NiO thin films with different annealing temperatures as a function of wavelength.

The ΔOD of the thin film was calculated from the transmission spectrum and only films annealed at temperatures above 300 °C had electrochromic response. This can be explained from the test results of FTIR and XRD. Only after being sufficiently annealed can nickel acetate in the precursor be fully converted into nickel oxide to have an electrochromic response. Figure 6 shows the ΔOD—Wavelength variation curves of nickel oxide films with different annealing temperatures. As the annealing temperature increases, the ΔOD of the films decreases significantly. The films annealed at 300 °C exhibit better optical modulation properties than those annealed at 400 °C and 500 °C. The change trend of ΔOD is also consistent with the change trend of the optical band gap, indicating that the change of the band gap is related to the optical change of the NiO film.

**Figure 7:**
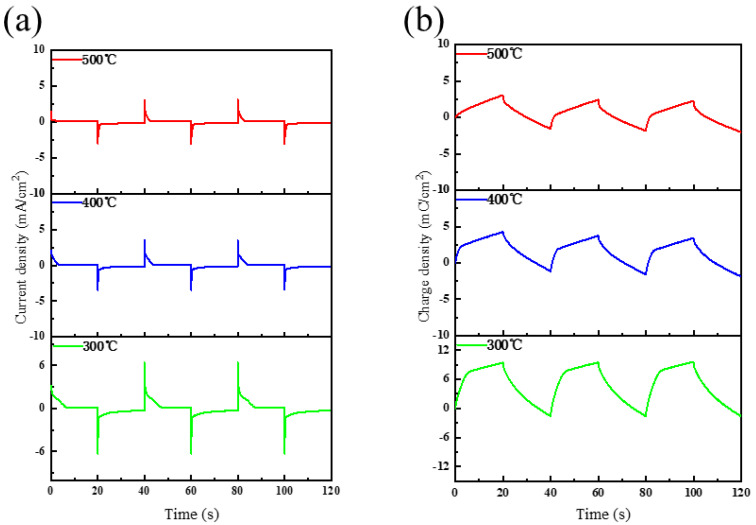
Current density curve (**a**) and charge density curve (**b**) of NiO films with different annealing temperatures.

The electrochemical performance of the film was also measured by an electrochemical workstation. Figure 7a shows the current density-time curves of NiO films with different annealing temperatures in three cycles. By integrating the curves, we can obtain the charge density-time curve (Figure 7b). With the increase in annealing temperature, the charge density in each electrochromic cycle of the film decreases significantly, which means that less electrochromic reactions occur, which can reasonably explain the aforementioned decrease in the light modulation ability of the film. 

**Figure 8:**
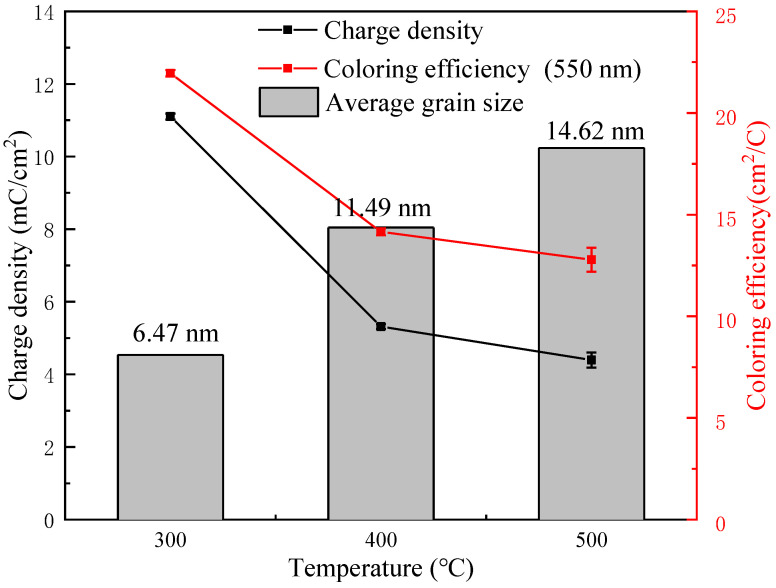
Trends in crystallite size, charge density, and coloring efficiency of NiO films with different annealing temperatures.

The coloring efficiency of the film at 550 nm was further calculated by Equation (4), so as to discuss the relationship between the crystallite size and coloring efficiency. Figure 8 shows trends in crystallite size, charge density, and coloring efficiency of NiO films with different annealing temperatures. According to the current research, some researchers believe that the electrochromic reaction of nickel oxide crystals is thought to occur inside the crystal crystallites [31], while other researchers believe that it occurs at the interface of the crystal crystallites [32]. If the electrochromic reaction of nickel oxide occurs at the crystallite interface, within the same unit volume, smaller crystallites have a larger surface area and can react with more ions, so they have a larger charge density. This means that more electrochromic reactions occur, that is, better optical modulation capabilities [33]. A larger surface area also means more efficient ion implantation, that is, higher electrochromic efficiency. By analyzing the change trend between the crystallite size, charge density, and electrochromic efficiency of NiO thin films, as the crystallite size increases, both the charge density and electrochromic efficiency decrease. The discoloration reaction is more likely to occur at the interface of the crystallites.

On the other hand, from the perspective of optical band gap analysis, the peak current density of these films in the coloring process decreased when the annealing temperature increased (a decrease from 6.2 mA/cm^2^ at 300 °C to 2.9 mA/cm^2^ at 500 °C). These were due to the decrease of *Eg* and the decrease of free electrons. Nickel oxide film with a smaller crystallite size has more electrons and ions implanted. When electrons and ions are injected into the nickel oxide film together, the same concentration of electrons and ions will be generated in the film, thereby reducing the optical band gap of the nickel oxide film. The reduction of the optical band gap increases the concentration of free carriers in the conduction band of nickel oxide, and the absorption and reflection of photons by free carriers causes changes in the optical properties of the film.

## 4. Conclusions

In this work, the effect of annealing temperature on the microstructure and performance of sol gel prepared NiO films were studied. When the annealing temperature is higher than 300 °C, the precursor nickel acetate is largely converted into nickel oxide with a considerable crystallinity and obvious electrochromic response. With the further increase of annealing temperature, the crystallinity film and crystallite size of NiO obviously increases. Analysis of the crystallite size, charge density, (∆OD), optical band gap, and coloring efficiency of films prepared under different annealing temperatures indicates that the electrochromic reaction of nickel oxide is more likely to occur at the crystallite interface, and smaller crystallite size is more conducive to ion implantation. Therefore, the nickel oxide explosion with a smaller crystal crystallite size can inject more ions, so that the optical band gap of the nickel oxide film is reduced, and more carriers are generated, which results in a stronger optical performance change. Under the premise of forming nickel oxide film, the reduction of annealing temperature is conducive to the formation of smaller crystallite size. The nickel oxide film annealed at 300° C has a small crystal crystallite size (6.47 nm), which is conducive to ion implantation to change the optical band gap and has good electrochromic properties.

## Figures and Tables

**Table 1:** Lattice parameters of NiO films in different directions at different annealing temperatures.

Annealing Temperature (°C)	a_(111)_ (nm)	a_(200)_ (nm)	a_(220)_ (nm)
300	0.4170	0.4166	0.4178
400	0.4164	0.4168	0.4171
500	0.4162	0.4167	0.4169

**Table 2:** Crystallite sizes of NiO films in different directions at different annealing temperatures.

Annealing Temperature (°C)	D _(111)_ (nm)	D _(200)_ (nm)	D _(220)_ (nm)
300	5.40	6.30	7.72
400	10.12	11.34	13.01
500	13.11	16.21	14.54
